# Relationship between serum total ige specific ige and serum total eosinophil counts in asthma bronchiale patients

**DOI:** 10.1186/1939-4551-8-S1-A25

**Published:** 2015-04-08

**Authors:** Fatma Nilufer Cakanlar, Erkan Ceylan, Asiye Kanbay

**Affiliations:** 1 Medeniyet University Goztepe Education Hospital, Turkey

## Background

Serum specific igE levels, serum total igE levels and serum total eosinophil counts in asthma bronchiale patients were compared with healthy controls and the relation between serum specific igE levels, serum total igE levels and serum total eosinophil counts were evaluated.

## Methods

5 specific igE level measurements (weed, 2 different tree panels [mixture of 5], feathers mix and dermatophagoides pterosyninus[Dp] and dermatophagoides farinae[Df]) and serum total igE levels, serum total eosinophil counts of a total 60 patients with Asthma (21 of them with asthma and allergic rhinitis) aged 19 to 52 (41 female, 19 male) and 34 (20 female, 14 male) healthy persons were compared.

## Results

60 percent of patient study group was determined to have positive specific igE, it is obtained that 70 percent of this positive specific igE group has sensitiveness to house dust mite antigen.

Asthma bronchiale cases have specific igE positiviness level and total igE level and total eosinophil counts were significantly higher than the control group.

Patients with asthma and allergic rhinitis revealed significant correlation with specific igE Df, total igE and eosinophil counts, furthermore significant correlation was behold between specific igE Dp, total igE and eosinophil counts.

## Conclusions

In our study compared to other allergens, sensitivity against house dust was much more higher and it is coherent with other studies in literature.

The usefullness of evaluating total serum igE or allergen specific igE for reasons of diagnosis and management is mutable however serum igE level is an important indicator of the intensity of inflammatory processes in allergic diseases and serum igE, total eosinophil counts and specific igE are helpful for diagnosis and developing therapeutic strategy in allergic diseases.

